# Machine Learning-Assisted Classification of Pathogenic Yeasts Using Laser Light Scattering and Conventional Microscopy

**DOI:** 10.3390/jimaging12030136

**Published:** 2026-03-19

**Authors:** Xiaoxuan Liu, Shamanth Shankarnarayan, Zexi Cheng, Manisha Gupta, Wojciech Rozmus, Mrinal Mandal, Daniel A. Charlebois, Ying Yin Tsui

**Affiliations:** 1 Department of Electrical and Computer Engineering, University of Alberta, Edmonton, AB T6G 1H9, Canada; xliu6@ualberta.ca (X.L.); zcheng5@ualberta.ca (Z.C.); mgupta1@ualberta.ca (M.G.); mmandal@ualberta.ca (M.M.); 2 Department of Physics, University of Alberta, Edmonton, AB T6G 2E1, Canada; sashanka@ualberta.ca (S.S.); wrozmus@ualberta.ca (W.R.); dcharleb@ualberta.ca (D.A.C.); 3 Department of Biomedical Engineering, University of Alberta, Edmonton, AB T6G 1H9, Canada

**Keywords:** machine learning, light scattering, pathogenic yeasts, label-free identification, microscopy image classification

## Abstract

Yeast infections are a major concern in clinical settings, and several known species are recognized for their antifungal drug resistance, especially the multidrug-resistant pathogen *Candidozyma auris*. It is of increasing importance to identify pathogenic yeasts to improve treatment outcomes. We present a technique to identify these yeast pathogens using machine learning with a neural network (DenseNet-201) on images obtained from laser light scattering and conventional microscopy. We performed the binary classification of seven species of pathogenic yeast based on their light scattering patterns and their microscopy images. We achieved an average classification accuracy of 95.3% for light scattering patterns and 96.6% for microscopy images of the yeast cells. We also demonstrate high classification accuracy when isolating *Candidozyma auris* images from all other species combined, at an average of 95.1% for light scattering patterns and 96.7% for microscopy images. The high average classification accuracies suggest that both light scattering and microscopy image data can be combined with machine learning models to classify pathogenic yeasts.

## 1. Introduction

Pathogenic yeasts are important contributors to invasive clinical infections and can be difficult to identify and treat. Invasive fungal infections affect over 2 million individuals worldwide, killing about 1.5 million people each year [1]. Unlike infectious bacteria and viruses, many pathogenic yeasts are part of the human microbiota and do not endanger human health until the individual becomes ill through other means, such as severe or chronic disease, injury or surgery, or while receiving immune-suppressing treatment [2]. Furthermore, many yeasts can survive in harsh environments and can easily spread from one location to another, leading to challenges in eliminating them in clinical settings. The use and misuse of antimicrobial drugs globally have raised concerns about pathogens developing drug, multidrug, or pandrug resistance [3], making it even more challenging to treat invasive fungal infections.

*Candida* species are the most common genus causing superficial and invasive fungal infections. The most common five *Candida* species that account for 90% of *Candida* infections worldwide are: *Candida albicans*, *Nakaseomyces glabrata* (previously *Candida glabrata*), *Pichia kudriavzevii* (previously *Candida krusei*), *Candida parapsilosis*, and *Candida tropicalis* [4]. Recently, *Candidozyma auris* (previously *Candida auris*), a multidrug resistant yeast, has emerged as an important pathogen with an alarming mortality rate up to 72% among candidemia patients [5,6]. Identifying this species is critical to deliver accurate and timely treatment to patients, as it has often been misidentified as *Candidozyma haemuli* (previously *Candida haemulonii*) and has the potential to cause hospital outbreaks [7,8]. However, current diagnostic methods to identify these yeast pathogens have long turnaround times.

Identifying fungal pathogens relies on conventional culture-based methods, matrix assisted laser desorption and ionization—time-of-flight mass spectrometry (MALDI TOF MS) and/or DNA sequencing [5,9]. However, most of these methods require sophisticated instrumentation and are lengthy processes that require days to complete. Furthermore, the lack of dedicated analysis/diagnostic laboratories within the hospital or clinical settings can cause patients to miss optimal treatment windows. Therefore, it is crucial to identify yeasts accurately within short periods of time using robust and accessible tools.

Many strategies are under development to investigate and identify pathogenic yeasts. Non-culture-based methods exist to identify systemic yeast infections, which cannot identify the infecting species. Furthermore, due to the low sensitivity and specificity of these tests they cannot be incorporated into routine diagnostics. Few studies have improved the molecular-based detection methods using variations of polymerase-chain reaction, but the false negative results, expensive nature, and multiple steps required restrict their use in diagnostics [10,11]. Genome sequencing methods exist to identify these fungi and investigate their virulence factors, but they are expensive and time-consuming [12,13]. Selective enrichment broths or agar media have also been investigated for the targeted screening of yeast species prior to other analyses [14,15].

Advancements in computing capabilities have enabled the development of machine learning (ML) techniques to conduct research in biological and medical fields [16,17,18]. Machine learning techniques have also applied to study pathogenic yeast species based on emerging diagnostic methods including microscopy images [19] and Raman spectroscopy [20]. Many of these diagnostic methods generate small-batch datasets which can be analyzed effectively through transfer learning [21]. In transfer learning, existing convolutional neural networks (CNNs) are trained on a dataset, typically a very large dataset for better generic learning, and then these trained networks are applied to another dataset for a related task.

Angular laser light scattering (ALLS) is a label-free method for studying cells. The method is rapid and requires only relatively simple sample preparation procedures. This method can be implemented in several different ways, such as using a sample cuvette [22,23] or microfluidics [24], and may be integrated in flow cytometer systems to take advantage of their existing complex functionality. While conventional microscopy typically observes cellular information on a single focal plane, light scattering-based approaches provide three-dimensional cellular information, such as the organelle distribution and membrane surface roughness [25,26]. This information is based on the differences in refractive index between different cellular components, which produce irregular interference patterns from the resulting scattered light. As such, irregular ALLS patterns, such as those deviating from alternating bands that are characteristic of homogeneous spheres, can indicate structural complexity of the cell. Previous studies [22,23,24,25,26,27,28] have used ALLS patterns to analyze and identify various cells (hematopoietic stem cells, Jurkat cells, THP-1 cells, SH-SY5Y cells, ovarian cancer cells, and cervical cancer cells). Machine learning techniques were also applied in several of these studies [22,28]. Compared to conventional microscopy, ALLS data is obtained without the need for optical focusing, resulting in fewer opto-mechanical components and less maneuvering of samples and their preparation, leading to low-cost robust equipment that has the potential for point-of-care implementation.

This paper presents a first study of using ALLS to investigate pathogenic yeasts, and the first systematic comparison of ALLS classification to conventional microscopy. Due to the alarming mortality rate of the multidrug-resistant *C. auris*, it is important to develop rapid detection tools for its presence in a sample. One key objective of our study is the detection of *C. auris* from other pathogenic yeasts in a shuffled image dataset. We propose a novel ML-based technique for the binary classification of pathogenic yeast species based on their light scattering patterns and microscopy images. Machine learning methods for machine vision typically require training a CNN to translate image data into a specific class. This training requires large datasets, possibly in the scale of millions of images; classification tasks with small datasets (<2000 images) may lead to inaccurate results. Therefore, in this study we employed transfer learning methods. Furthermore, we employed binary classification, which is also capable of achieving good performance on small datasets and is suitable for our goal of isolating *C. auris* from the other yeast species. We challenged our method by applying it to light scattering patterns of pathogenic yeast species that are morphologically similar to each other. We achieved a high binary classification performance of cell species at high cell concentrations for improved data throughput, and successfully classified *C. auris* from other species. In general, we find that ML models trained in light scattering or microscopy image data can be used to quickly and accurately identify pathogenic yeasts.

## 2. Materials and Methods

**Figure 1:**
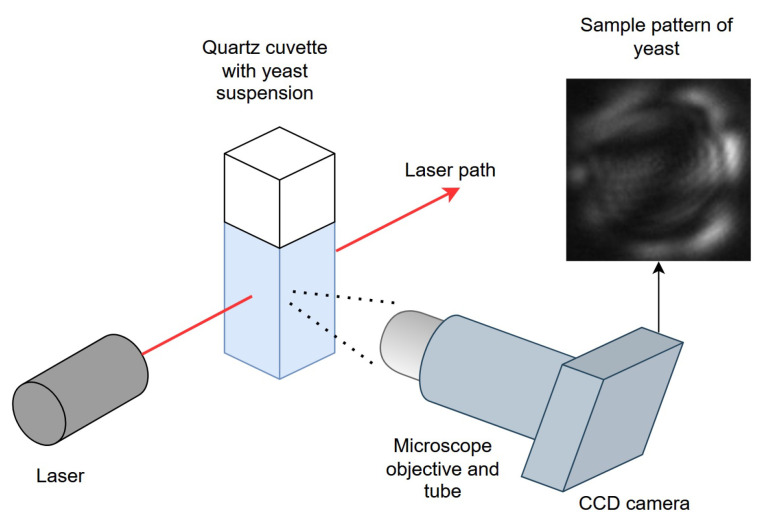
Schematic diagram of the benchtop experimental setup for light scattering pattern collection.

The light scattering setup consists of a helium-neon laser (1 mW nominal power, Melles Griot), which was focused to 0.6 mm diameter to visualize light scattering events. A quartz macro-cuvette with 3 mL capacity was used to hold the yeast suspension. A charge-coupled device (CCD) monochrome camera (CMLN-13S2M-CS, Point Grey Research, Richmond Canada) equipped with a microscope objective (10× magnification, 0.25 NA) and 20 cm lens tube was placed at 90° with respect to the laser path to collect light scattering events. All data collection was carried out in the dark. The working distance of the objective was adjusted until light scattering events are defocused to fill the height of the detector (Figure 1). The half-cone angle of the microscope objective centered at 90° from the laser direction was approximately 11°, corresponding to an angular range of 79°–101°.

A procedure reported previously [23] was followed to confirm the experimental setup was working properly. Briefly, polystyrene microbeads with 6 μm mean diameter (Polysciences, Cat. No. 07312, Warrington, PA, USA) diluted to 3000 beads/mL were placed in the macro-cuvette to visualize their light scattering pattern as single events. The light scattering pattern image, which consists of light and dark fringes, was observed from the experimental setup. It was compared to an image from Mie scattering simulation of a spherical particle with 6 μm diameter for an angular range of 79°–101° and with refractive index = 1.5875. The simulated image consists of 5 light fringes agreeing well with the experimental observation indicating the setup is working properly.

**Figure 2:**
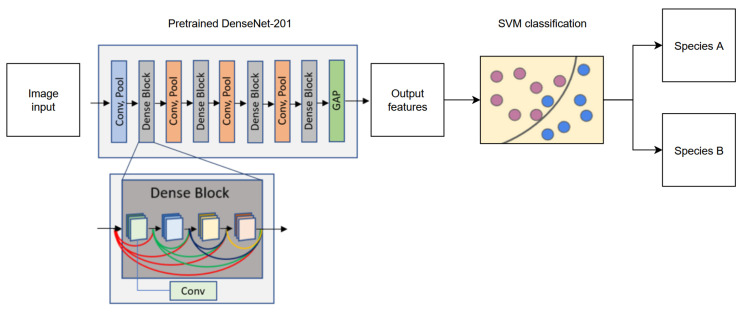
Schematic diagram of the transfer learning algorithm using a pre-trained CNN (DenseNet-201) and an SVM to perform the binary classification of pathogenic yeast species. Within a dense block, each Conv layer receives features from all preceding layers in the same block, from left to right. Feature propagation is arbitrarily colored to represent origin from a different layer in the same block. Pink and blue circles represent arbitrary features of species A and species B mapped in feature space by the SVM.

Schematic representation of our proposed transfer learning method is provided in Figure 2. From each image, 1920 features were extracted using a pre-trained DenseNet-201 [29] model implemented in PyTorch 1.3.1. In DenseNet-201, each Dense Block consists of multiple layers where the inputs are outputs from every preceding layer in that block, thus improving feature reuse and mitigating the vanishing gradient problem, the latter of which is particularly important, as the images of the yeast cells can appear very similar. Each Dense Block is preceded by a convolution and a pooling layer. It is worthwhile to mention that fine-tuning the fully connected layer in this model did not achieve satisfactory performance after training for 20 epochs on a preliminary task with the same dataset. After global average pooling (GAP), the fully connected layer is removed to record the resulting 1920 features from each image for a species. DenseNet-201 is frequently used for classification tasks in biology and medicine [30,31,32,33]. This model had already been trained on ImageNet-1K data for general purpose usage, and no further modifications were made to the neural network. For binary classification, features from any two species were then passed to a radial-basis function (RBF) kernel support vector machine (SVM) with 5-fold cross-validation and parameter C = 1 [34]. This parameter modulates between decision plane margin size and training error, where a small C allows for large margins and a large C limits the SVM to fewer misclassifications. C was selected based on good performance when employed in our previous work [22]. Compared to a linear-kernel SVM [35], the RBF SVM maps features into high-dimensional space and is particularly useful when the relationship between the label and features is nonlinear or unknown as was the case for our study. The SVM was trained to find a hyperplane that best separates the features from the two classes. Features from images in the testing group for the two species were then passed to the SVM to separate them using the hyperplane.

All images (light scattering or microscopic) were resized to 224 × 224 pixels and normalized to [0, 1] to ensure consistent quality before the ML method was applied. To preserve directional information of the light scattering patterns and for equality, data augmentation was not used in all experiments. The algorithm was evaluated on a machine with Ryzen 5 7600X 6-Core Processor, 4.70 GHz CPU (Advanced Micro Devices, Santa Clara, CA, USA), GeForce RTX 4060 GPU (Nvidia, Santa Clara, CA, USA), and 32 GB RAM.

The protocols for culture of pathogenic yeast were described previously [19]. Briefly, four species of yeast (*C. albicans*, *C. auris*, *N. glabrata*, and *C. haemuli*) from clinical samples were obtained from the Alberta Precision Laboratories—Public Health Laboratory and three yeast species [*C. parapsilosis* (ATCC 22019), *P. kudriavzevii* (ATCC 6258), and *C. tropicalis* (ATCC 750)] were obtained from American Type Culture Collection (ATCC). All yeast isolates were stored in 25% glycerol at −80 °C. Before each experiment, yeast cells were cultured from frozen stock onto Sabouraud Dextrose Agar (SDA) plates (Merck KGaA, Darmstadt, Germany) and incubated at 35 °C for 48 h and further sub-cultured onto new SDA plates at 35 °C for 24 h before light scattering and microscopy image data collection. The cells were then harvested into normal saline (0.9%) from the culture dishes, and the optical density (0.09–0.13) was measured at 530 nm in a spectrophotometer (Voriskan Lux, Thermofisher Scientific, Singapore VL0000D0). This corresponds to approximately 1–5 × 10^6^ cells/mL. Furthermore, cell samples were diluted in phosphate buffered saline until 3000 cells/mL were obtained. During light scattering pattern collection, samples were mixed with a micropipette at 10 min intervals to avoid cells settling.

**Figure 3:**
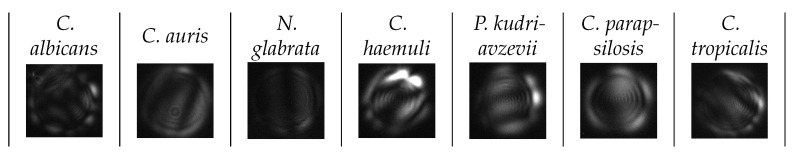
Light scattering patterns collected from the 7 species of pathogenic yeast. Typical image size is 250 × 250–500 × 600 pixels.

A total of 1532 light scattering patterns across 7 species of pathogenic yeasts were obtained from the light scattering classification experiment (Table 1). Light scattering patterns were obtained from captured video frames above the background intensity threshold (typical value at 18 on a scale of 0–255) and cropped manually to remove as much background as possible around the circular pattern boundary to remove background artifacts (Figure 3). Patterns that were too small (<60 × 60 pixels) or too large (>600 × 600 pixels) were removed, as they were too near or too far from the detector to show a discernible pattern.

**Figure 4:**
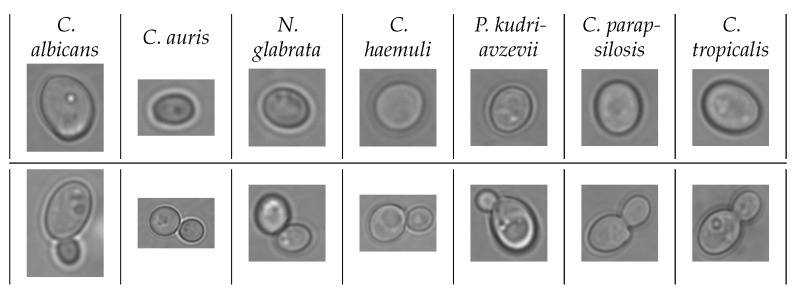
Microscopy images from 7 species of pathogenic yeast. Top row: single cells; bottom row: budding cells. Data obtained from [19,40]. Typical image size is 250 × 250–500 × 600 pixels.

The yeast cell suspension to obtain microscopy images was prepared similarly to that of the light scattering experiments. After measuring the cells in the spectrophotometer, 40 μL of the suspension was placed on a glass slide (Fisher Brand, Cat. No. 22034486, Pittsburg, PA, USA) and covered with a coverslip (Fisher Brand, Cat. No. S17525C, Pittsburg, PA, USA). Then, the microscopy images were collected in brightfield mode using EVOS M7000 (Invitrogen, Thermo Fisher Scientific, Waltham, MA, USA) at 100× (oil immersion). A total of 100 whole images were obtained for each of the 7 yeast species. Then, a total of 1400 (200 for each species) microscopy images of single cells and 1400 microscopy images of budding cells across 7 species of pathogenic yeasts were cropped out from the whole images for the microscopy classification experiment (Table 2). Cropping was done around the boundary of the cells, reserving approximately 20 pixels on each side to avoid over-cropping due to defocusing of the lens. Representative microscopy images of single cells and budding cells from each of the seven yeast species are provided in Figure 4.

## 3. Performance Evaluation

Prior to applying the proposed model, the light scattering and microscopy image datasets were verified by Gradient-weighted Class Activation Map (Grad-CAM) to generate heatmaps showing regions of interest for the ML model (Supplemental Materials Figures S1–S21) [41]. Visual inspection was used to ensure the regions of interest were located on light scattering patterns or microscopy images of yeast cells. For heatmaps of ALLS patterns, regions of high interest tended to be brighter than surrounding areas or had very distinct contrast, indicating significant structures in or on the cell, such as budding growth or large organelles like the nucleus. For microscopy images, heatmaps showed interest where clusters of organelles were distinctly visible. Furthermore, the heatmaps of microscopy images of budding yeast tended to highlight the budding site where the cells are attached, indicating the model’s recognition of the growth.

The proposed model was evaluated in three different ways. First, we evaluated the binary classification of all the laser scattered images. Then, we performed the binary classification on the microscopy images. Finally, we performed the binary classifications of *C. auris* versus all the non-*C. auris* light scattered images and conventional microscopy images. Here, *C. auris* was used as the focal species, as this pathogenic yeast species is of high interest due to its recent spread across the globe and multidrug-resistant nature. We report the performance of our ML models as classification accuracy (ACC), which is the ratio of total correct predictions (including true positives (TP) and true negatives (TN)) to total classification predictions.

### 3.1. Binary Classification–Laser Scattered Images

Binary classifications with 5-fold cross-validation were performed with 150 randomly selected images from each class. Every classification was performed between two classes, where images from the two classes were separated by the SVM decision hyperplane and the number of testing images correctly and incorrectly classified were recorded for performance evaluation. Each class was classified against every other class in iterative fashion (e.g., *C. albicans* versus *C. auris*, then *C. albicans* versus *N. glabrata*, and so on) resulting in six classifications for each class. However, classifications such as *C. albicans* vs. *C. auris* are the same as *C. auris* vs. *C. albicans*, so the total number of unique classifications was the number of combinations of two classes selected from seven classes, or 7!/[2!(7−2)!]=21.

For 5-fold cross-validation, the 150 images from each of the seven classes were divided into five non-overlapping subsets of 30 images each. For each fold, four out of five subsets were used for training the SVM decision plane, and the remaining subset was used for testing. The testing subset was changed for each of the five folds. The testing classification ACC of each classification was averaged after 5-fold cross-validation.

### 3.2. Binary Classification—Microscopy Images

For each species in the microscopy images, the images were also separated by type: single cell (SC), budding cell (BC), and combined. In the combined case, all SC and BC images of a single species were grouped together. Binary classification of seven species of yeast with 5-fold cross-validation was performed as described in Section 4.1 for all SC, BC, and combined cases.

### 3.3. Binary Classification—*C. auris* Versus Non-*C. auris* Species (Laser Scattered and Microscopy Images)

To perform the binary classification of a one-versus-all scenario for *C. auris*, the images of the other species (*C. albicans, N. glabrata, C. haemuli, P. kudriavzevii, C. parapsilosis*, and *C. tropicalis*) were assembled into one class and dubbed “*Others*”. Features for each image belonging to either class of *C. auris* or *Others* were extracted and classified as described in Section 3 and Section 4.1.

### 3.4. Evaluation Indicators

For light scattering and microscopy classification of each yeast species, only the accuracy is reported for brevity. For the *C. auris* versus non-*C. auris* case, we evaluated the performance based on the accuracy (ACC), sensitivity (SEN, also called the true-positive rate TPR), precision (PRE), specificity (SPE), area under the receiver operating characteristic (ROC) curve (AUC), and F_1_ score. These metrics are defined, using true positive (TP), true negative (TN), false positive (FP), and false negative (FN):

(1) $$\begin{array}{c} \begin{array}{c} ACC=\frac{TP+TN}{TP+FP+TN+FN}\; \end{array} \end{array}$$

(2) $$\begin{array}{c} \begin{array}{c} SEN=\frac{TP}{TP+FN}\; \end{array} \end{array}$$

(3) $$\begin{array}{c} \begin{array}{c} PRE=\frac{TP}{FP+TP}\; \end{array} \end{array}$$

(4) $$\begin{array}{c} \begin{array}{c} SPE=\frac{TN}{FP+TN}\; \end{array} \end{array}$$

(5) $$\begin{array}{c} \begin{array}{c} AUC=\int_{0}^{1}TPR\left(x\right)\;dx\; \end{array} \end{array}$$

(6) $$\begin{array}{c} \begin{array}{c} F_{1}=\frac{2TP}{(2TP)+FP+FN}\; \end{array} \end{array}$$

Here, x denotes the value of the false positive rate, defined as FPR=FP⁄((TN+FP)).

## 4. Results and Discussion

### 4.1. Light Scattering Images

We employed ML methods to classify images obtained from two different methods to differentiate clinically important yeast species. The binary classification accuracies of the seven pathogenic yeast species are shown in Table 3, and the calculated average ACC is 95.31%. ACC over 95% were found in 12 out of 21 unique binary classification cases and included all seven species tested, indicating that all species possess distinguishing light scattering features that can highly separate them from at least one other species. ACC over 90% were found in 18 out of 21 unique cases, suggesting the presence of distinct optical features that could exist for all species investigated.

**Figure 5:**
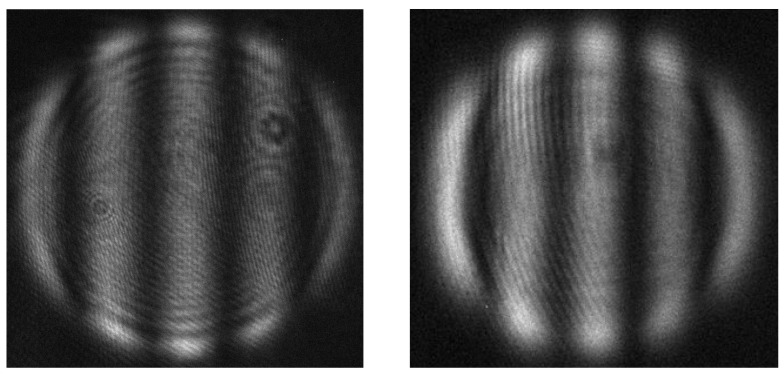
Comparison of an experimental light scattering pattern of polystyrene sphere (**left**) to a representative light scattering pattern of *N. glabrata* (**right**).

*N. glabrata* had the most cases of ACC greater than 95% at 5 out of 6, and 94.33% when classified against *P. kudriavzevii*. This shows that the light scattering patterns of *N. glabrata* were the most distinct out of all 7 species tested. *C. auris* had four out of six cases where ACC was higher than 95%, also suggesting that its light scattering patterns were highly distinguishable from most other species. The structural and optical properties of the yeast cells impact the resulting light scattering images. These properties include the cell size, cell shape, organelle distribution, and cell surface and cell wall structure, as well as the refractive index differences between the cellular components. *N. glabrata* was the only species that had straight, fringe patterns, which is indicative of a smooth, homogeneous sphere (Figure 5). Similar straight fringes were observed in a previous batch of data collected from *N. glabrata* as well, though the number of fringes were different, indicative of a different cell size (see Supplemental Materials Figure S22). In the current study, 54% of the *N. glabrata* images consisted of fringe patterns, which explain its high distinction from other species. This also suggests that *N. glabrata* had much higher tendency to grow into a morphology that optically resembled a homogeneous sphere, though further analysis of specific optical features is required to investigate this. The drastic difference in cell size of *C. auris* likely contributes to its high ACC when classified against *C. albicans*, *N. glabrata* (in addition to its spheroidicity), *C. parapsilosis*, and *C. tropicalis*. Cell size and shape differences may be more obvious when using light scattering, as *C. auris* also had ACC near 94% when classified against *C. haemuli*, in contrast to their phylogenetic similarity [7]. The same ACC was found when classifying *C. auris* vs. *P. kudriavzevii*, indicating the presence of optical features that may be useful for isolating this species from others.

For all binary classifications, the lowest ACC was found when classifying *C. parapsilosis* from *C. tropicalis* (82.67%), indicating the highest degree of confusion between them among all species tested. This suggests similarity between the two species in terms of optical properties at the single-cell level that could lead to the overlap in the feature space, such as similar shape and internal structure. ACC of *C. albicans* versus *C. haemuli* and versus *C. parapsilosis* were also relatively low, at around 86% and 88%, respectively, which indicates a degree of confusion among these three species as well.

### 4.2. Microscopy Images

The ML-based binary classification technique was also applied to microscopy images for budding cells, single cells, and both combined (Table 4). The calculated average accuracies are 96.66%, 97.15% and 96.55% for single cells (SC), budding cells (BC), and combined, respectively. The results were significant, with *p*-values of the average accuracies all below 0.01 under the null hypothesis of an ACC of 50%. For the combined case, images of single and budding cells from each species were pooled together before feature extraction and classification for better comparability to the light scattering image performance. In 3 out of 21 unique classification cases, the difference between SC ACC and BC ACC was 5% or higher, suggesting that there is a benefit of investigating SC and BC of these cases separately. Out of 21 unique cases, 17 cases had SC ACC above 95%, 16 cases had BC ACC above 95%, and 16 had combined ACC 95% or above. Like the light scattering results, ACC at 95% or above included all species, indicating that they all possess visually distinct features for classification.

The lowest ACC from these classification pairs is between SC of *C. parapsilosis* and *C. tropicalis*, at 62.67%, indicating high confusion between these two species when observing only SC. This classification outcome is also the only one for microscopy images where the ACC is low, 12% above 50%, which is the threshold for ML random guessing, and further alludes to the similarity between the SC of these two species. Interestingly, when classifying only the BC case for *C. parapsilosis* and *C. tropicalis*, the ACC is 97.67%. This suggests that while the individual SCs may show similarities, once they begin to reproduce the two species are immediately discernible. This may be due to different growth characteristics such as the relative size, shape, and visibility of organelles in the daughter cell. Visual inspection of the SC and BC images revealed that *C. parapsilosis* mother cells tended to grow into elongated shape when budding (16% of budding images had mother cell length over twice its width), while *C. tropicalis* mother cells were generally rounder (1% of budding images were elongated). SC of both species were inspected for shape as well: 5% of *C. parapsilosis* and 2% of *C. tropicalis* were elongated cells. The combined case ACC of *C. parapsilosis* vs. *C. tropicalis* was 79.67%, and the calculated average of the SC and BC ACC to 80.17%, with 0.5% difference. However, the combined case did not always yield better performance, as the combined cases’ ACC for *C. albicans* vs. *N. glabrata* (92.67%), *P. kudriavzvii* vs. *C. parapsilosis* (92%), and *P. kudriavzevii* vs. *C. tropicalis* (93.67%) were all lower than or equal to their respective SC (93.33%, 97%, and 98%) and BC (97%, 92.33%, and 93.67%) cases, respectively. Additionally, the averaged ACC of these combined cases were also lower than both their SC and BC cases, albeit only by 0.11%. This implies that classification of SC and BC separately provides benefit for these species pairs, while for most of classification cases using the combined case was sufficient to achieve high ACC. Subtle differences in performance between single-cell and budding-cell classifications may reveal shifts in overall population uniformity when yeast cells are growing. This can be seen in the cases of *C. auris* vs. *C. haemuli* and of *N. glabrata* vs. *C. haemuli*, where the ACC of classifying budding cells (92.16% and 90.33%, respectively) is lower than the ACC of single cells (98.67% for both species pairs), indicating potential similarities in growth behavior between these species pairs. The opposite was observed for *C. albicans* vs. *C. auris* and *C. albicans* vs. *N. glabrata*, suggesting that classifying them based on their budding yields better results. The ACC of all other species pairs were higher than or equal to 90.33%, suggesting visual differences between them, such as in their shape and organelle visibility and distribution.

### 4.3. Comparing Light Scattering Image and Microscopy Image Performance

A single-sample *t*-test was performed for the light scattering classifications and all the microscopy image classifications, namely SC, BC, and combined. The null hypothesis stated that the proposed ML was unable to classify any species in this study, with an average ACC of 50% across 21 unique binary classification cases that corresponded to a random-guess classifier. The *p*-values of average ACC for light scattering and for all microscopy image cases were all found to be <0.0001. The results were found to be significant at *p* < 0.05.

Since the light scattering setup does not distinguish SC and BC, the ACC of classification of light scattering images was viewed together with only the combined case from microscopy images (Table 5). For 8 out of 21 species pairs, the ACC difference between light scattering images and microscopy images were higher than 5%, with the greatest ACC difference at 11% for the *C. albicans* vs. *C. haemuli* classification. The comparative results remain inconclusive as to which method demonstrates superior performance; the calculated average ACC for all species pairs for light scattering and microscopy were 95.31% and 96.55%, respectively. Furthermore, no single method outperforms the other across all species pairs, indicating that the optical properties from light scattering and the visual properties from microscopy of these yeasts are not directly associated. Agreement between both methods on overall classification ability for the seven yeast species is also shown in the unique case of *C. parapsilosis* vs. *C. tropicalis*, where both image types had the lowest ACC among all species pairs and their ACC matched closely with only 3% difference (79.67% to 82.67%). Obtaining lower classification ACC in the light scattering results for *C. albicans* vs. *C. haemuli* agrees with previous findings using an InceptionV3 model, where the precision for these two classes was also found to be the lowest when trained on a larger set of yeast image data [19]. This further suggests that while the two methods searched for different characteristics of the yeasts, they arrived at similar conclusions about the segregation of these species, which could be beneficial for cross-validation purposes.

### 4.4. Classification of *C. auris* Versus All Other Species

To test the transfer learning method’s ability to discriminate *C. auris* from the other six species of yeast, a one-versus-all binary case was implemented using 150 light scattering patterns from *C. auris* and *Others* and the results are shown in Table 6 for the average of 5-fold cross-validation.

The average classification ACC was over 95%, suggesting that the method was adequate when the other classes are combined. The computation time taken for the one-versus-all case is provided in Table 7. Using the proposed method, the computation time scales linearly with the amount of data provided to the feature extractor and only needs to be performed once. Meanwhile, each run of the SVM binary classification can be kept to approximately 2 min when selecting 150 images from each class. Thus, high performance can be achieved using relatively short time and with small datasets.

A similar evaluation was performed on all microscopy images of seven yeast species, and the performance is provided in Table 8. The performance of the transfer learning method was higher by 1.6% in average ACC and F_1_ score of microscopy images than light scattering images, which may be attributed to the larger dataset size. Overall, the analysis performance agrees between the two datasets and shows that this analysis method might be applicable to other population studies where the isolation of one species is of high priority.

A single-sample *t*-test was also performed for the one-versus-all cases of both the light scattering classification and microscopy image classification. The null hypothesis stated that the classifier was unable to classify *C. auris* and the Others groups, with an average ACC of 50% in five runs, corresponding to each of the five folds. The *p*-values for both light scattering and microscopy image classifications were <0.0001, and the results were found to be significant at *p* < 0.05.

In this study, we classified seven species of pathogenic yeast using a combination of binary classification and transfer learning on their light scattering patterns and microscopy images. The high binary classification ACC of *C. auris* versus each other six species resulted in high binary classification of *C. auris* versus all six species together. Since the seven species studied presently account for more than 90% of Candida infections, we expect high binary classification ACC of *C. auris* versus all other yeast species that cause Candida infections. In conventional statistics, when two independent detection methods are used, such as light scattering and microscopy, the overall probability of both methods incorrectly classifying the sample is smaller than that of each method alone. In general, classification accuracy and classification probability have a positive linear relationship [42]. Thus, it is reasonable to expect that the confidence for detection of *C. auris* can be improved by using both light scattering and microscopy methods compared to using either method alone. Furthermore, the combination of the two methods can also improve prospects of applying the classification to real clinical samples, where pathogenic yeast cells may be lower in number and where other cells, such as blood cells, or other microbes may be present. Nevertheless, as we have demonstrated that pathogenic yeasts can be classified on a species-level with high accuracy, we may anticipate that the classification of these yeasts versus other cells that are significantly different from them can be promising.

## 5. Conclusions

Our study highlights the potential of using both light scattering and conventional microscopy methods to classify pathogenic yeasts in label-free conditions. For the first time, pathogenic yeasts were studied based on their light scattering patterns. We classified seven species of pathogenic yeasts using a combination of binary classification and transfer learning on their light scattering patterns and microscopy images. We have also demonstrated for the first time a systematic comparison of light scattering classification with microscopy image classification. We found that while *C. auris* and *C. haemuli* are frequently misidentified due to their phylogenic similarity [7], our method can discern them with ACC above 92% using either light scattering or conventional microscopy data. Meanwhile, the spread of light scattering pattern classification ACC for other species pairs was found to be uneven. Specifically, *C. albicans* and *C. haemuli* were found to be most likely to be misclassified as other species, whereas *C. tropicalis* was found to be most likely misclassified as *C. parapsilosis*. We also show for the first time the identification of *C. auris* from other species through our binary classification approach, resulting in an average ACC of 95.1% and 96.7% for light scattering patterns and conventional microscopy images, respectively. The similarity in performance of light scattering images and microscopy images demonstrates that the transfer learning approach is adequate for classifying image types, and that comparing the results of both methods in parallel can improve diagnostic confidence. The classification accuracies of microscopy images of single cells, budding cells, and combination of both image types were nearly identical. This suggests that the features of the species themselves are sufficient for classification, which agrees with a recent study that used explainable AI to determine the image features (organelle, cell interior, cell wall, budding patterns/scars, and optical patterns) that advanced computer vision models use to classify fungal pathogens [40]. Interestingly, each of these methods reveal different characteristics about the cell. From light scattering images we found that *N. glabrata* was unique among the seven species to produce patterns that resemble homogeneous spheres, and this characteristic may be useful to rapidly screen *N. glabrata* and research its internal structure. Light scattering patterns are also sensitive to the cell size, a feature which may be lost during pre-processing of conventional microscopy images, such as through cropping and resizing target cells. On the other hand, microscopy images could be further categorized as single cells or budding cells, which light scattering is incapable of, and this had a great impact on the classification of microscopy images, such as in the case of *C. parapsilosis* versus *C. tropicalis*. This further illustrates the potential benefits of identifying pathogenic yeasts using a combination of features from light scattering and microscopy images. Future research involves the development of a dual-modality model for integrating light scattering and microscopy data, such as using light scattering for initial screening and microscopy for in-depth cell visualization and characterization. Our proposed transfer learning classification of yeast species is applicable to the fast classification of other cell types or image types with low computation costs, opening the possibility for clinical diagnostic use.

## Figures and Tables

**Table 1:** Number of images of light scattering patterns collected from each species of pathogenic yeast.

Type	Number of Images	Cell Size
*C. albicans*	278	4–10 μm [36]
*C. auris*	218	2–3 μm [37]
*N. glabrata*	156	1–4 μm [36]
*C. haemuli*	314	2–3 μm [38]
*P. kudriavzevii*	182	2.2–15.2 μm [39]
*C. parapsilosis*	222	2.5–9 μm [36]
*C. tropicalis*	162	4–11 μm [36]
Total	1532	

**Table 2:** Number of single-cell microscopy images from each species of pathogenic yeast. Data obtained from [19,40].

Type	Single Cell	Budding Cell
*C. albicans*	200	200
*C. auris*	200	200
*N. glabrata*	200	200
*C. haemuli*	200	200
*P. kudriavzevii*	200	200
*C. parapsilosis*	200	200
*C. tropicalis*	200	200
Total	1400	1400

**Table 3:** ACC of binary classification of light scattering patterns of 7 pathogenic yeast species. Classifications between *C. auris* and other species are highlighted in green. Classifications between *C. haemuli* and other species are highlighted in blue. Classification between *C. auris* and *C. haemuli* is highlighted in darker green. Classifications between two identical species are highlighted in black.

	*C. albicans*	*C. auris*	*N. glabrata*	*C. haemuli*	*P. kudriavzevii*	*C. parapsilosis*	*C. tropicalis*
*C. albicans*							
*C. auris*	98.33						
*N. glabrata*	99.33	96.67					
*C. haemuli*	86.00	93.67	98.00				
*P. kudriavzevii*	99.33	93.67	94.33	97.33			
*C. parapsilosis*	88.00	99.00	100.00	93.00	99.67		
*C. tropicalis*	90.67	99.33	99	93.67	100.00	82.67	

**Table 4:** Accuracy of binary classification of microscopy images of 7 pathogenic yeast species. The cell entries are in X/Y/Z format to denote the accuracies for single cells/budding cells/single and budding combined, respectively. Classifications between *C. auris* and other species are highlighted in green. Classifications between *C. haemuli* and other species are highlighted in blue. Classification between *C. auris* and *C. haemuli* is highlighted in darker green. Classifications between two identical species are highlighted in black.

	*C. albicans*	*C. auris*	*N. glabrata*	*C. haemuli*	*P. kudriavzevii*	*C. parapsilosis*	*C. tropicalis*
*C. albicans*							
*C. auris*	93.00/96.00/96.67						
*N. glabrata*	93.33/97.00/92.67	93.00/92.33/92.00					
*C. haemuli*	99.67/96.67/97.00	98.67/92.16/97.33	98.67/90.33/95.00				
*P. kudriavzevii*	99.33/98.33/98.67	99.67/99.33/98.67	99.00/100.00/99.67	100.00/100.00/100.00			
*C. parapsilosis*	99.67/98.33/98.33	99.33/99.33/98.33	99.33/99.33/100.00	100.00/99.33/100.00	97.00/92.33/92.00		
*C. tropicalis*	100.00/99.67/100.00	100.00/98.67/99.33	99.67/99.67/99.00	100.00/100.00/99.67	98.00/93.67/93.67	62.67/97.67/79.67	

**Table 5:** ACC of light scattering image classification (X) and ACC of microscopy image classification with combined case only (Y) in X/Y format. Classifications between *C. auris* and other species are highlighted in green. Classifications between *C. haemuli* and other species are highlighted in blue. Classification between *C. auris* and *C. haemuli* is highlighted in darker green. Classifications between two identical species are highlighted in black.

	*C. albicans*	*C. auris*	*N. glabrata*	*C. haemuli*	*P. kudriavzevii*	*C. parapsilosis*	*C. tropicalis*
*C. albicans*							
*C. auris*	98.33/96.67						
*N. glabrata*	99.33/92.67	96.67/92.00					
*C. haemuli*	86.00/97.00	93.67/97.33	98.00/95.00				
*P. kudriavzevii*	99.33/98.67	93.67/98.67	94.33/99.67	97.33/100.00			
*C. parapsilosis*	88.00/98.33	99.00/98.33	100.00/100.00	93.00/100.00	99.67/92.00		
*C. tropicalis*	90.67/100.00	99.33/99.33	99.00/99.00	93.67/99.67	100.00/93.67	82.67/79.67	

**Table 6:** Performance metrics of the classification of *C. auris* versus *Others* for 5 runs using light scattering images.

	ACC	SEN	PRE	SPE	AUC	F_1_
Average	95.07	95.00	94.95	94.93	98.60	0.95

**Table 7:** Time taken to perform feature extraction of two classes of light scattering data (*C. auris* and *Others*) and classification tasks using the computation resources reported.

Feature Extraction per Image for *C. auris*	Feature Extraction per Image for *Others*	Testing Time per Image
0.35 s	0.35 s	0.42 s

**Table 8:** Performance metrics of the classification of *C. auris* versus *Others* for 5 runs using microscopy images.

	ACC	SEN	PRE	SPE	AUC	F_1_
Average	96.73	95.73	97.69	97.73	99.59	0.97

## Data Availability

Data underlying the results presented in this paper are not publicly available at this time due to privacy but may be obtained from the authors upon reasonable request. The code used for this study can be found at: https://github.com/xxliu-ua/Yeast_2026/tree/main, accessed on 18 February 2026.

## Supplementary Materials

The following supporting information can be downloaded at: https://www.mdpi.com/article/10.3390/jimaging12030136/s1, Figures S1–S7: Representative light scattering patterns of 7 yeast species (left) with their respective heatmaps (right); Figures S8–S14: Representative single cell microscopy images of 7 yeast species (left) with their respective heatmaps (right); Figure S15–S21: Representative budding cell microscopy images of 7 yeast species (left) with their respective heatmaps (right); Figure S22: Representative fringe light scattering pattern of *N. glabrata* from a duplicated experiment.

## Abbreviations

The following abbreviations are used in this manuscript:

MALDI TOF MS	Matrix-assisted laser desorption and ionization—time-of-flight mass spectrometry
DNA	Deoxyribonucleic acid
ML	Machine learning
CNN	Convolutional neural network
ALLS	Angular laser light scattering
CCD	Charge-coupled device
RBF	Radial-basis function
SVM	Support vector machine
ATCC	American Type Culture Collection
SDA	Sabouraud dextrose agar
GRAD-CAM	Gradient-weighted class activation map
ACC	Accuracy
TP	True positive
SC	Single cell
BC	Budding cell
SEN	Sensitivity
TPR	True positive rate
SPE	Specificity
AUC	Area under the curve
ROC	Receiver operating characteristic
TN	True negative
FP	False positive
FN	False negative
