# Supplementary material for: Machine Learning-Assisted Classification of Pathogenic Yeasts Using Laser Light Scattering and Conventional Microscopy
Source: J Imaging. 2026 Mar 19;12(3):136. doi: 10.3390/jimaging12030136 (PMC13027885; doi:10.3390/jimaging12030136)
Supplement: Supplementary file 1 [file jimaging-12-00136-s001.zip › jimaging-4187230-supplementary.pdf]

Supplementary Materials: Machine Learning-Assisted Classification of Pathogenic Yeasts Using Laser Light Scattering and Conventional Microscopy

Xiaoxuan Liu [1], Shamanth Shankarnarayan [2], Zexi Cheng [1], Manisha Gupta [1], Wojciech Rozmus [2], Mrinal Mandal [1], Daniel A. Charlebois [2,3] and Ying Yin Tsui [1,*]

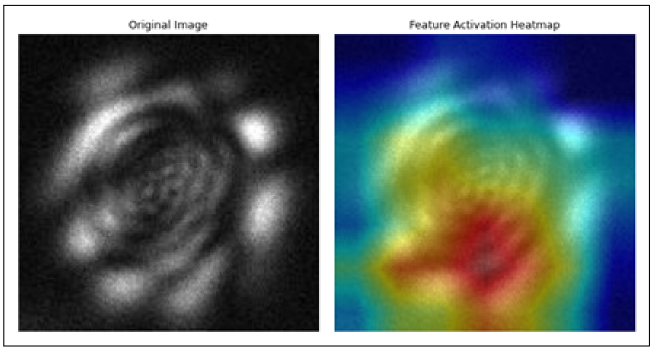

Figure S1. Representative light scattering pattern of *C. albicans* (left) and its heatmap overlay (right).

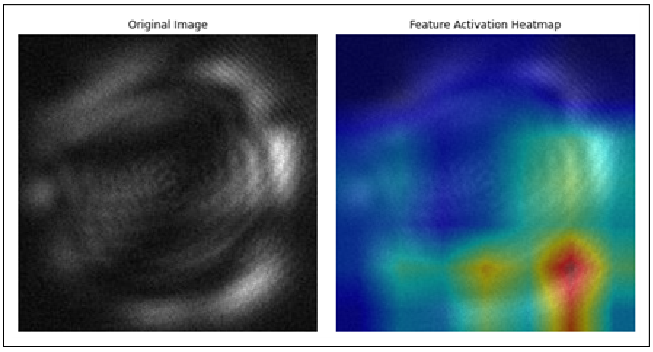

Figure S2. Representative light scattering pattern of *C. auris* (left) and its heatmap overlay (right).

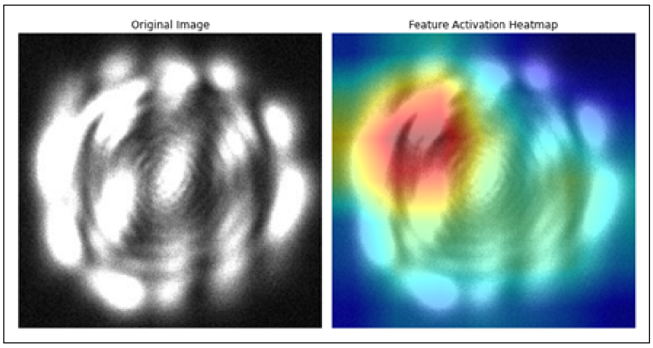

Figure S3. Representative light scattering pattern of *N. glabrata* (left) and its heatmap overlay (right).

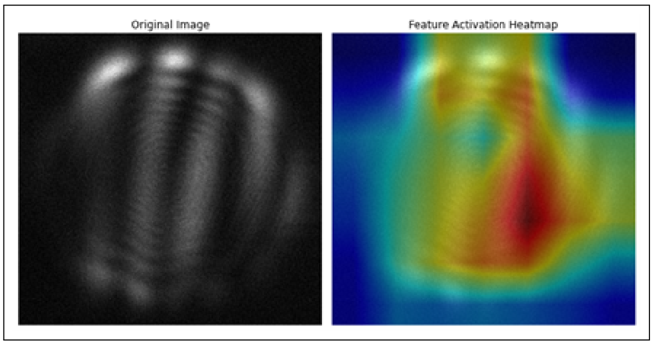

Figure S4. Representative light scattering pattern of *C. haemuli* (left) and its heatmap overlay (right).

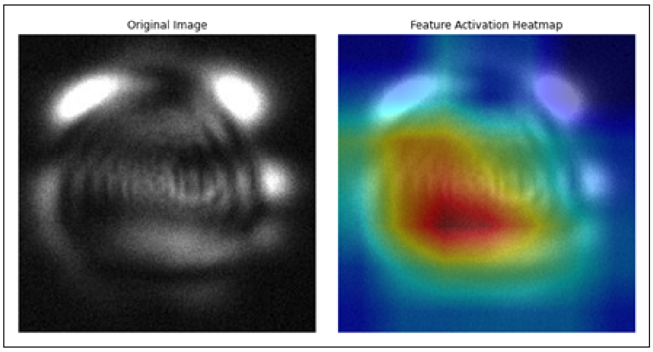

Figure S5. Representative light scattering pattern of *P. kudriavzevii* (left) and its heatmap overlay (right).

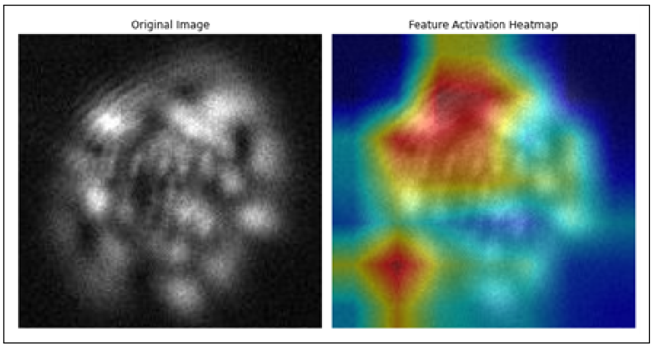

Figure S6. Representative light scattering pattern of *C. parapsilosis* (left) and its heatmap overlay (right).

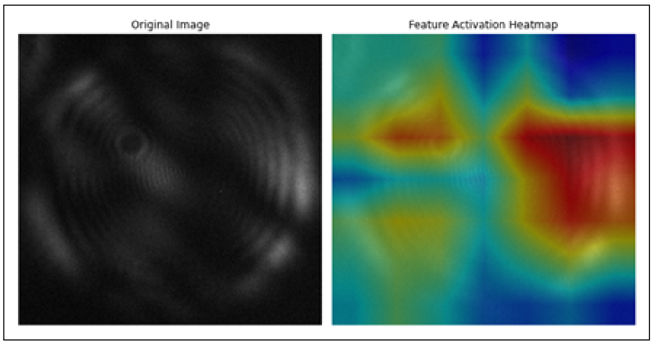

Figure S7. Representative light scattering pattern of *C. tropicalis* (left) and its heatmap overlay (right).

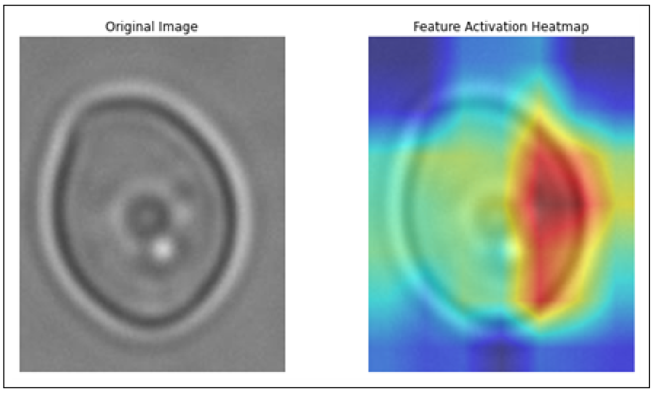

Figure S8. Representative single cell microscopy image of *C. albicans* (left) and its heatmap overlay (right).

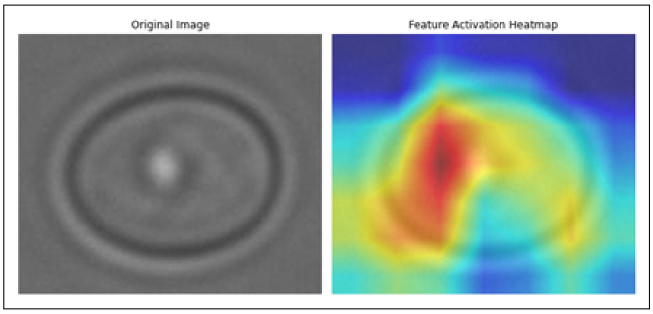

Figure S9. Representative single cell microscopy image of *C. auris* (left) and its heatmap overlay (right).

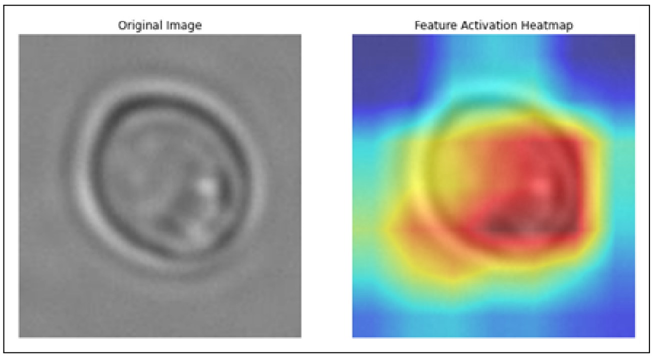

Figure S10. Representative single cell microscopy image of *N. glabrata* (left) and its heatmap overlay (right).

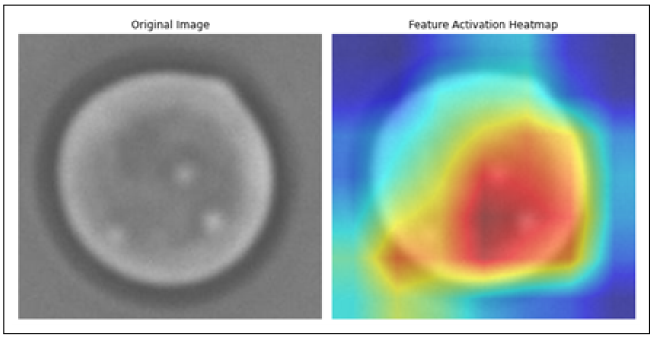

Figure S11. Representative single cell microscopy image of *C. haemuli* (left) and its heatmap overlay (right).

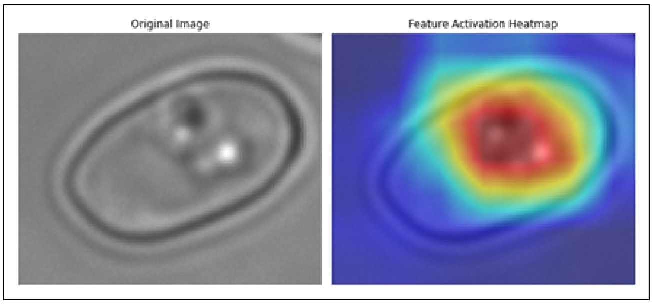

Figure S12. Representative single cell microscopy image of *P. kudriavzevii* (left) and its heatmap overlay (right).

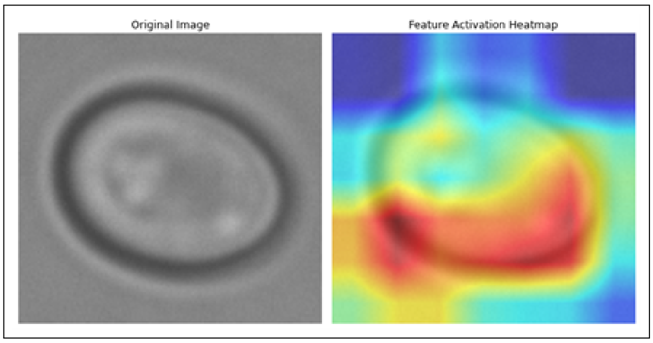

Figure S13. Representative single cell microscopy image of *C. parapsilosis* (left) and its heatmap overlay (right).

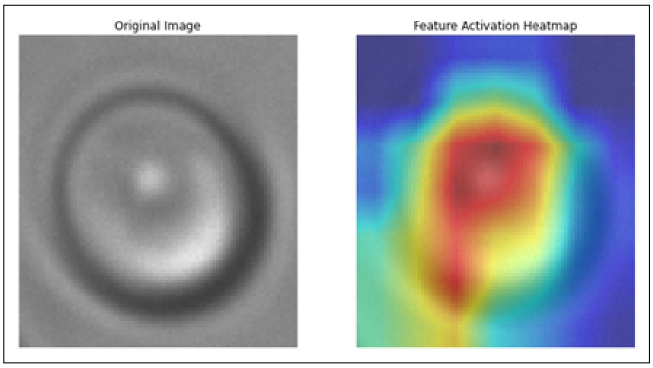

Figure S14. Representative single cell microscopy image of *C. tropicalis* (left) and its heatmap overlay (right).

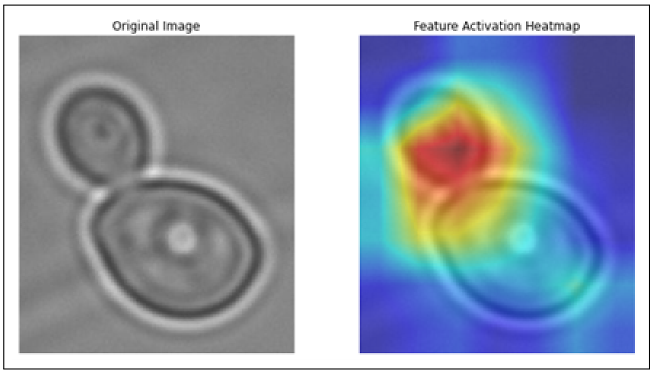

Figure S15. Representative budding cell microscopy image of *C. albicans* (left) and its heatmap overlay (right).

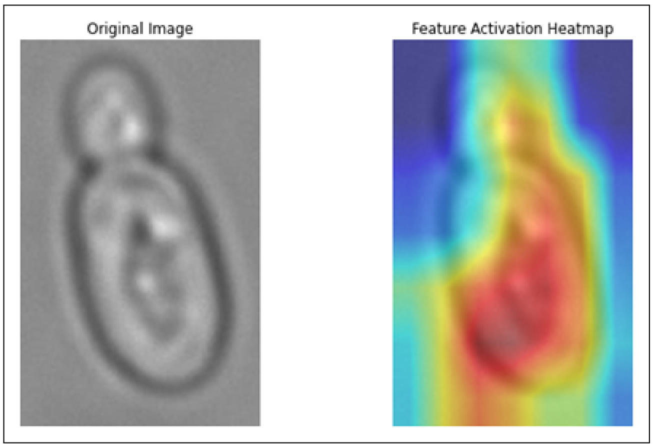

Figure S16. Representative budding cell microscopy image of *C. auris* (left) and its heatmap overlay (right).

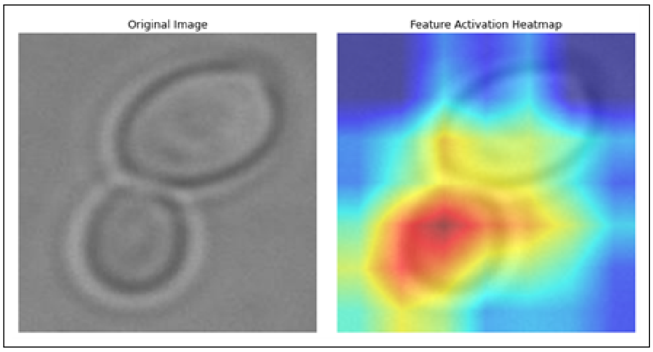

Figure S17. Representative budding cell microscopy image of *N. glabrata* (left) and its heatmap overlay (right).

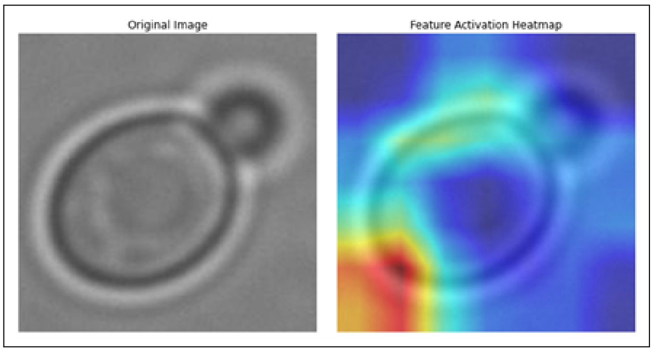

Figure S18. Representative budding cell microscopy image of *C. haemuli* (left) and its heatmap overlay (right).

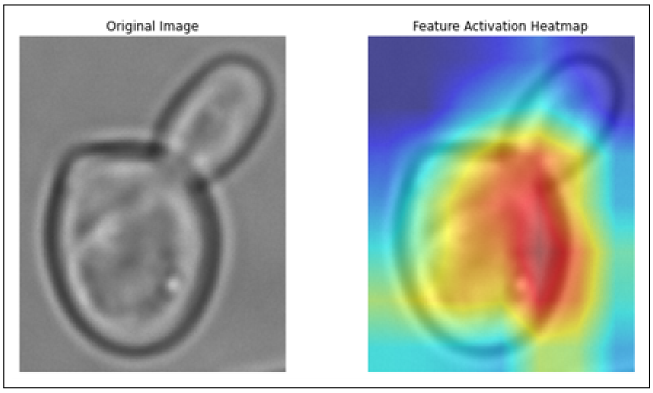

Figure S19. Representative budding cell microscopy image of *P. kudriavzevii* (left) and its heatmap overlay (right).

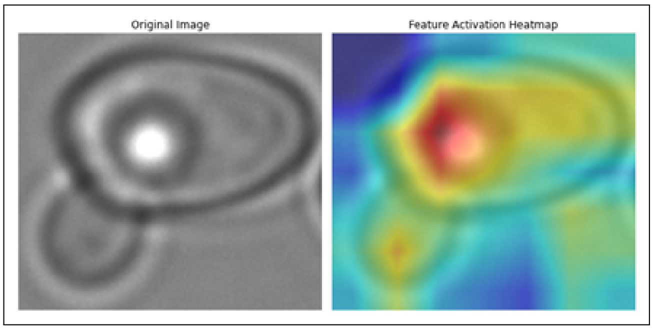

Figure S20. Representative budding cell microscopy image of *C. parapsilosis* (left) and its heatmap overlay (right).

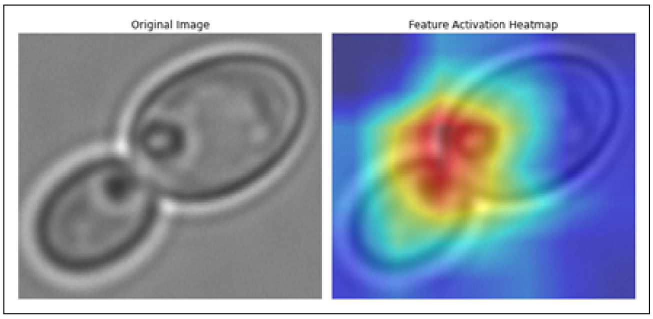

Figure S21. Representative budding cell microscopy image of *C. tropicalis* (left) and its heatmap overlay (right).

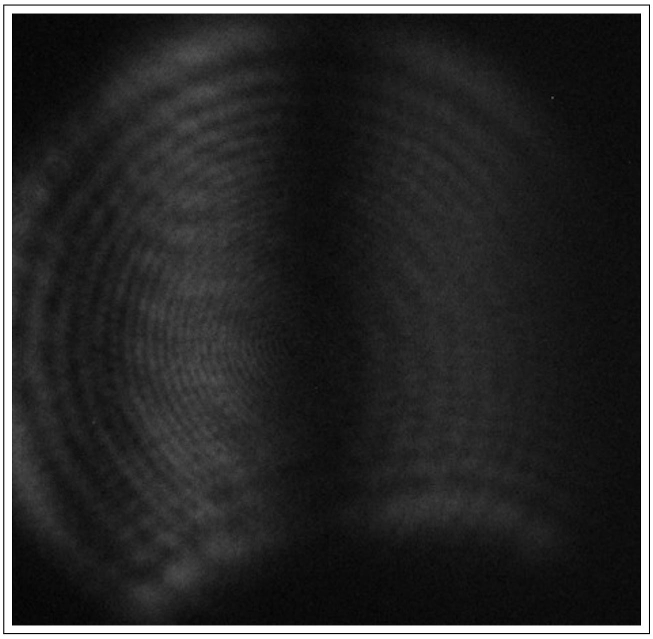

Figure S22. Representative fringe light scattering pattern of *N. glabrata* from a duplicated experiment.
